# Attitudes and readiness of nurses towards digitalisation

**DOI:** 10.4102/curationis.v48i1.2706

**Published:** 2025-05-30

**Authors:** Calvin Mabaso, Ramasela Mokonyama, Jeremy Mitonga-Monga

**Affiliations:** 1 Department of Industrial Psychology and People Management, College of Business and Economics, University of Johannesburg, Johannesburg, South Africa

**Keywords:** digitalisation, nurses’ attitudes, reading, electronic medical records, perceptions

## Abstract

**Background:**

This study examines nurses’ attitudes and readiness to integrate digital technologies, particularly electronic medical records (EMR), in a South African private hospital group. Understanding these perspectives is vital as healthcare increasingly digitises.

**Objectives:**

The research aimed to assess the state of readiness of healthcare professionals before the implementation of an integrated EMR system within a multidisciplinary private hospital environment.

**Method:**

A qualitative exploratory design was used, with semi-structured interviews conducted with 15 participants. The data were thematically analysed, reaching saturation at 14 participants.

**Results:**

Six key themes emerged: effective communication, implementation reasons, training support, overall employee perceptions, technology literacy and limitations of EMR. The study highlights the need for targeted interventions to bridge knowledge gaps and foster a supportive environment for digital healthcare integration.

**Conclusion:**

The shift from manual to electronic health records is crucial for improving efficiency, patient care and sustainability in healthcare. However, this transition requires careful consideration of human factors, such as behaviour, attitudes and readiness.

**Contribution:**

The study underscores the need for a strategic approach to technology adoption, emphasising the benefits of EMR implementation, tailored training and continuous communication, alongside addressing emotional support, technology literacy and network stability.

## Introduction

Information and communication technologies (ICTs) are increasingly employed in developed and developing nations to enhance accessibility, efficiency and productivity within healthcare systems (Qureshi, Ahmad & Nawaz 2012). One such advancement is the electronic medical record (EMR) system, which has emerged as a critical solution to modernise healthcare management (Coleman, Herselman & Potass 2012). According to the International Telecommunications Union (ITU), integrating EMRs into healthcare systems improves patient outcomes by streamlining information management and reducing medical errors. Electronic health records (EHRs) and EMRs are often used interchangeably but have distinct functions. Electronic health records collect longitudinal patient data across multiple healthcare providers, supporting interoperability and coordinated care (Kim et al. 2019). In contrast, EMRs are typically restricted to a single healthcare organisation, focusing on digital patient care documentation within that setting. Despite these distinctions, both technologies enhance healthcare efficiency and decision-making.

The Fourth Industrial Revolution (4IR) has accelerated digitalisation across various industries, including healthcare. Digitalisation extends beyond digitisation, where paper records are merely converted into electronic formats to involve fundamental changes in service delivery through integrated digital systems (Asare, Otoo-Arthur & Frimpong 2017; Clarke 2019). The World Economic Forum (2019) notes that digitalisation has driven global demand for quality healthcare systems, prompting many countries to adopt EMRs to improve healthcare administration, patient management and research (Fraser et al. 2005). Despite the clear benefits of EMRs, their adoption in developing countries remains slow because of financial constraints, technological limitations and organisational resistance (Biruk et al. 2014). For example, attempts to implement national EHR systems in Malawi and Ghana were hindered by inadequate infrastructure, unreliable electricity and healthcare professionals’ (HCPs) reluctance to adopt digital tools (Katurura & Cilliers 2018). In South Africa, multiple EHR systems from different vendors have led to database incompatibilities, preventing seamless communication between healthcare facilities. Over 50% of public health centres still rely on paper-based filing systems, highlighting the need for a more integrated approach to digital healthcare.

Successful digital transformation in healthcare is not solely dependent on technological infrastructure; it also requires HCPs to be ready and willing to adopt new tools. Technology readiness refers to an individual’s propensity to embrace and use new technologies to achieve professional goals (Kuo et al. 2013). Burke and Weill (2009) argue that information technology rapidly expands in healthcare, encompassing EMRs, telemedicine and AI-driven decision support systems. However, studies indicate that resistance to digital transformation remains a significant barrier. Chengoden et al. (2023) found that concerns about data security, privacy and inadequate training hinder digital adoption among healthcare workers. Similarly, Tack et al. (2022) surveyed over 1000 HCPs, revealing persistent reluctance, despite recognising the advantages of digital tools. Organisational culture plays a crucial role in addressing these barriers. Kinicki and Fugate (2021) emphasise that strong leadership and a supportive environment are essential for successful digital adoption. Lampo (2022) further highlights the importance of clear guidance and innovation champions in promoting digital transformation. Hyde et al. (2023) found that involving HCPs in shaping digital strategies enhances their willingness to embrace change.

Given these insights, this study explores HCPs’ readiness for an integrated EMR system in a private hospital setting. The research aims to identify key enablers and barriers influencing digital transformation by assessing technical and cultural factors. While many studies highlight the general challenges of EMR adoption, a gap exists in understanding the specific complexities private hospitals face in developing countries. This study seeks to bridge that gap by offering insights into how digital transformation can be effectively implemented in healthcare settings.

### Aim and objectives

The study aimed to assess the state of readiness of HCPs before implementing an integrated EMR system within a multi-disciplinary private hospital environment. This study’s objective was to assess nurses’ perceptions, attitudes and level of readiness for adopting an integrated EMR system. In addition, it aims to identify key challenges and barriers influencing nurses’ acceptance and use of the system.

### Literature review

#### Technology acceptance model and theory of change behaviour

New technologies have been continuously adopted within the healthcare sector to enhance the quality of patient care (Blackwell & Blackwell 2008). Pena-Lopez (2010) supports this view, noting that the latest technologies specifically improve service delivery in clinical and hospital settings. These advancements contribute to patient safety, staff efficiency and effectiveness while helping to reduce organisational expenses (Scott 2008). However, adopting technology in healthcare also presents several challenges, including readiness and attitudes towards using technology. Davis’ (1989) technology acceptance model (TAM) will be explored to address these challenges. The TAM focuses on understanding individual intentions to use new technology by examining perceived usefulness and ease of use (Davis 1989). According to Abdullah, Ward and Ahmed (2016) and Scherer et al. (2018), these two factors explain the behavioural intention to accept and use new technology. Tubaishat (2018) emphasises that perceived usefulness is particularly significant in explaining technology acceptance among nurses.

Perceived usefulness is the degree to which an individual believes a particular technology will enhance job performance (Marangunić & Granić 2015). This enhancement is vital for improving healthcare services in hospitals. Conversely, perceived ease of use refers to how effortless an individual believes using the technology will be (Davis 1989). This factor predicts a user’s acceptance or rejection of technology. Petty (2012) adds that attitudes towards the system – considering its benefits in improving work performance and efficiency – play a crucial role in technology acceptance. These elements help the healthcare sector gauge the acceptance of new technologies. This study focuses on healthcare workers who may perceive new technology as challenging or a waste of time, potentially hindering its adoption. Conversely, if healthcare workers view new technology as stimulating and easy to learn, they are more likely to embrace it. Strudwick (2015) further argues that applying a modified TAM tailored to the healthcare context can better explain nurses’ technology acceptance. Proper technology training can facilitate the acceptance of new technologies among healthcare workers. Ahlan and Ahmad (2015) also report that understanding healthcare workers’ perceptions, attitudes and readiness through such models is valuable.

#### Innovation diffusion theory

Innovation diffusion theory (IDT) is a sociological framework that explains how new ideas, products or technologies spread within a society. Jillbert et al. (2023) emphasise that IDT focuses on adopters and communication channels, identifying stages of adoption ranging from innovators to laggards (Goh & Sigala 2020). In the context of healthcare, digital technologies and systems are viewed as innovations undergoing an adoption process, where healthcare workers can be categorised as innovators, early adopters, early majority, late majority and laggards (Putteeraj et al. 2022). Understanding these categories provides hospital groups with critical insights into the attitudes and readiness of healthcare workers towards digitalisation (Jillbert et al. 2023).

The healthcare sector has undergone significant digital transformation in recent years to enhance service delivery. Johansson et al. (2017) argue that digitalisation and the integration of information technology (IT) are vital for addressing modern healthcare challenges. Currie and Finnegan (2009) further highlights that incorporating ICT in healthcare reduces costs and improves quality and efficiency, leading to higher patient satisfaction. However, Yusuf, Rahman and Subiyakto (2024) caution that adopting IT disrupts existing processes and workforce structures, requiring strategic organisational changes for effective implementation.

Given the disruptive nature of digitalisation, change management is crucial in healthcare transformation. The Health Information Management Systems Society (2014) defines change management in healthcare as the integration of project management and human behaviour strategies to achieve digital transformation goals on time and within budget. Zandieh et al. (2008) argue that resistance to change is a major barrier to successful innovation adoption, making stakeholder engagement essential. Benham-Hutchins (2009) reinforces this point by asserting that effective digital transformation requires structured change management strategies, including active participation of HCPs, during the implementation process. Despite the benefits, digitalisation in healthcare remains challenging (Yusif, Hafeez-Baig & Soar 2020). Yarbrough and Smith (2007) notice that resistance among healthcare workers – often because of negative attitudes and a lack of readiness – frequently leads to failures in digitalisation initiatives. However, Lackner (2015) asserts that successful digital adoption depends on behavioural adaptation among stakeholders, ensuring that digital transformation aligns with organisational culture and workflow dynamics.

#### Attitude towards organisational change

Traditionally, hospitals have relied on manual methods to coordinate and maintain health records. However, in response to the evolving healthcare landscape, many hospitals are now undergoing a significant shift towards adopting EHR systems. Electronic health records are digital representations of patient data, providing immediate and secure access exclusively to authorised individuals (Katuu 2015). Both public and private hospitals, as well as healthcare providers, are increasingly integrating technology into their operational frameworks. This technological adoption aims to enhance patient care, improve care quality, reduce errors and increase overall efficiency (Luxford, Safran & Delbanco 2011). Factors beyond the technological aspects influence the decision to adopt EHR systems. Significant changes in business processes, clinical workflows and daily tasks are also required (Martin & Voynov 2014). Often, organisations underestimate the importance of change management when implementing EHRs. This is frequently reflected in limited user involvement, which is typically restricted to the operational phase (Schmucker 2009). Effective implementation and operation of EHR systems demand a well-planned change management approach. Integrating change management into the overall strategy is essential, as it provides valuable insights into how individuals can prepare for, acquire the necessary tools for and support each other in the implementation process. Change management is recognised as a discipline that helps organisations to achieve successful outcomes by ensuring that everyone is adequately prepared and engaged (Prosci n.d.).

#### Readiness towards organisational change

Readiness plays a crucial role in determining employees’ initial support for change initiatives (Holt et al. 2007). Although the concept of readiness was first introduced by Jacobson (1957), it has since been expanded upon in various theoretical frameworks to understand the complexities of the change process better. Van de Ven and Poole’s (1995) seminal work synthesises change theories from multiple disciplines, offering a valuable framework for researchers and practitioners in organisational development. Organisational leaders often implement systemwide changes to achieve specific objectives: a concept known as teleological change (Van de Ven & Poole 1995). These intentional modifications can lead to tensions between leaders and organisation members. For change to succeed, it is essential to address conflicts and align beliefs and cognitions between organisational leaders and members. This process is called dialectical change (Van de Ven & Poole 1995). Establishing readiness is vital for successful change implementation. As a result, assessing readiness before initiating changes has become a common practice, leading to the development of various evaluation tools (Cunningham et al. 2002; Jones, Jimmieson & Griffiths 2005; Weeks et al. 2004). These tools assess readiness from multiple perspectives: the change process, the content of change, the context of change and individual attributes (Holt et al. 2007). In organisational dynamics, the change process involves the systematic steps taken during implementation. A key dimension within this process is the level of employee participation. The content of change refers to the specific initiative being introduced, such as administrative, procedural, technological or structural changes. The organisational context encompasses the environmental factors influencing employees and their behaviours. A learning organisation is characterised by its employees’ ability to embrace and adapt to ongoing changes. Finally, individual attributes highlight variations among employees in their willingness to accept and support organisational change.

#### Nurses’ attitudes and readiness for digitalisation

Nurses’ attitudes towards digitalisation are significantly influenced by their understanding and knowledge of new technology. For technology to be effectively adopted, it must be accepted and engaged with by employees (Niedzwiecka & Pan 2017). Introducing new technology requires careful management, thoughtful change management strategies and attention to employee well-being. Technology can create unease about job security, especially among less educated employees, while those familiar with artificial intelligence and robotics may have more positive views. Digitalisation transforms industries, including healthcare, by changing working conditions and reducing the need for physical presence. Although technology offers many benefits, such as increased efficiency and new opportunities, it also requires employees to learn new skills, leading to increased unemployment for specific job roles. Kolade and Owoseni (2022) state that digitalisation is causing mass unemployment resulting from computers replacing jobs at a much faster rate than people can acquire new skills. The shift towards digital platforms changes work descriptions and labour legislation as remote work and online platforms become more prevalent. Recent studies, such as Chengoden et al. (2023), have examined healthcare workers’ readiness to embrace digital tools. Tack et al. (2022) surveyed over 1000 HCPs to assess their attitudes and readiness for digital transformation. Despite recognising the benefits of digital tools for patient care and administrative efficiency, the study highlighted significant concerns, including data security, privacy issues, a lack of training and resistance to change. To support healthcare workers adapting to digital advancements, targeted training programmes and robust infrastructures are necessary.

## Research methods and design

### Research design

A qualitative, exploratory research design was employed to explore the nurse’s attitudes and readiness towards digitalisation and usage of an electronic medical recording system in the private healthcare sector. Saunders, Lewis and Thornhill (2019) explain that exploratory research design is a valuable approach for posing open-ended enquiries aimed at uncovering the intricacies of a particular subject and obtaining insights about it. Qualitative researchers are enabled to understand issues by examining how they are contextualised by individuals and by considering the meanings that they attach to them (Creswell & Miller 2000).

### Setting

This research study was conducted within a private healthcare hospital group operating in different regions across South Africa. Initially, a diverse range of hospitals varying in size, location (urban) and speciality was considered. However, the final participant selection focused on a single hospital, with 15 participants drawn from all 8 departments, allowing for an in-depth exploration of HCPs’ attitudes and readiness towards the integrated EMR system. The study was conducted in the context of an EMR system that was in the process of being introduced. While the research primarily assessed readiness before implementation, participants also reflected on their experiences during the system’s rollout. This setting allowed researchers to capture a wide range of perspectives and experiences among healthcare workers in different contexts.

### Population and sampling

This study’s target population is comprised of 410 employees from a private healthcare organisation. Only employees who have been employed for 2 years or more were selected to participate in the study. Because of their exposure to the work environment, their responses to the research may be more accurate and reliable. In this study, the researcher used purposive sampling to select the individuals for the study. This method identifies the individuals who possess in-depth knowledge of the given research subject (Shaheen & Pradhan 2019). Fifteen participants were selected from all eight departments. During the interviews, the researcher consistently asked questions sequentially reflected in the interview schedule until data saturation was reached (Merriam & Tisdell 2016). The researcher interviewed healthcare workers who responded to the invitation email, which was emblematic of their willingness to be involved in the study until the data saturation point was reached during the 14th interview. Data were collected through in-depth interviews, providing rich qualitative insights into the participants’ experiences and perspectives.

### Introduction to establishing the researcher’s role

Before accessing the research setting, the researcher liaised with the gatekeepers (hospital managers) to conduct research within the organisations. My research role was congenially related to the participants as a custodian of their perspectives and experiences (Esterberg 2002). It accomplished this by acting ethically and expressing genuine interest in the research participants.

### Data collection methods

The researcher conducted face-to-face semi-structured interviews. The interviews were conducted at a time and day convenient for the participants and held at their work premises at the hospital. Before the interviews, the participants were contacted to confirm their availability and the scheduling of suitable meeting times. Each interview took about 30–90 min.

### Data analysis

To organise, structure and derive meaning from the collected data (De Vos et al. 2011), the analysis began with the transcription of all participant interviews. This study employed an inductive approach to data analysis, as it focused on reducing the volume of raw information and identifying significant patterns, as suggested by Henning, Van Rensburg and Smit (2004). Inductive analysis allowed for themes and insights to emerge from the data rather than being guided by predefined theories or frameworks. Qualitative interview data were analysed manually using thematic analysis, following Braun and Clarke’s (2013) guidelines. This approach facilitated the identification of key themes and patterns across the dataset, ensuring a structured and systematic analysis to address the research questions. According to Kiger and Varpio (2020), thematic analysis is a process that uses qualitative data collected from various sources to identify common themes and ideas in a set of texts. It involves closely examining the data to identify the patterns and themes of meaning that emerge repeatedly. The six steps of thematic analysis were used as a guideline (Peel 2020). The data analysis process began with *data familiarisation*, where the researcher immersed themselves in the data by reviewing transcripts multiple times to gain a deep understanding of the content. This was followed by *initial coding*, where significant data segments were identified and labelled with codes representing key ideas or features. Next, *grouping codes* involved organising similar codes into broader categories, ensuring alignment with the research questions. These categories were then clustered into overarching themes in the *theme development* stage, capturing the essence of the data. The themes were further refined in the *theme refinement* phase to ensure coherence and alignment with the dataset and study objectives. Finally, in the *validation and finalisation* stage, themes were cross-checked to confirm their relevance and distinctiveness.

### Trustworthiness of research

Trustworthiness is explained as the ability to be honest, dependable and reliable (Kaur et al. 2022). The concept is measured by implementing credibility, transferability, confirmability and dependability. Credibility was achieved through probing to ensure sufficient data and prolonged engagement with participants. The researcher carried out some member checks to ensure that there were no prejudices or errors. Dependability was achieved as the data were recorded and the transcripts were kept and available upon enquiry. Confirmability was achieved in this study, as member reflections were used to support the analysis’s credibility, which means that participants were asked to comment and elaborate on early findings. In addition to saturation, data sufficiency was also used to indicate trustworthiness, implying that the study was adequately designed to identify all the key attributes needed to answer the study question. To ensure transferability, participants were chosen for the study carefully; they were cited directly from the data; and the research context and participants were thoroughly described.

### Ethical considerations

Ethical clearance to conduct this study was obtained from the University of Johannesburg Department of Industrial Psychology and People Management (IPPM) Research Ethics Committee (No. IPPM-2022-693(M)). Ethical considerations were carefully integrated into this study to ensure the protection and rights of participants. Prior to data collection, participants were fully briefed on the study’s purpose, procedures, and potential risks. Informed consent was obtained without coercion, with participants marking a symbol on the interview guide to indicate voluntary agreement. They were assured that non-participation would have no negative consequences. Confidentiality was ensured by anonymising identities and securely storing data. The study was mindful of potential power dynamics, creating a respectful environment where participants could speak freely. Ethical approval was secured in accordance with institutional guidelines to uphold research integrity.

## Results

### Demographic characteristics of participants

Fifteen participants working at the private hospital group were interviewed, and the job categories of the participants were unit managers, registered nurses, enrolled nurses and enrolled nursing assistants. The participants were drawn from different units or wards of the hospital, namely, the Neonatal ICU, Orthopaedic Surgical Ward, Medical Ward, Cardiothoracic ICU, Trauma High Care Unit, Neuro ICU, General ICU and Theatre. Regarding classification, these participants work in large hospitals with multiple disciplines. The employees who participated in the study were both males and females whose ages ranged between 26 and 64. The five unit managers’ qualifications are Bachelor of Nursing Science (Education and Administration, Post-Basic qualification in the following specialities – Neonatal ICU, Trauma, Neuro and Operating Theatre. The 10 Registered Nurses qualifications are Bridging II and post-basic qualifications in the following speciality – Cardiothoracic, Orthopaedics and General ICU.

### Thematic analysis of the collected data

Based on the responses from the research participants, the following six themes emerged regarding attitudes and readiness to implement digitalisation in private healthcare hospital groups: effective communication, implementation reasons, training and support, overall perception of employees, technology literacy and limitation of the whole EMR approach. Table 1 presents the themes and sub-themes derived from the thematic data analysis.

**TABLE 1:** Themes and sub-themes identified through thematic data analysis of interview transcripts.

Theme	Sub-theme
1. Effective communication	1.1 Breakdown communication
2. Rationale for EMR system implementation	-
3. Training support	-
4. Overall perception of employees	-
5. Technology literacy	-
6. Limitations of the whole-EMR approach	-

EMR, electronic medical records.

### Theme 1: Effective communication

The theme was developed when the participants were asked if the EMR system was communicated to them before implementation. Ten participants (100%) confirmed that there was clear communication regarding implementing the EMR system. The management and project team ensured that the necessary and relevant information was communicated prior to the implementation of the system:

‘Received a lot of communication from hospital management about the project prior to the roll-out.’ (P6, female, enrolled nurse)

‘Communication about the project began well in advance of implementation.’ (P8, female, registered nurse)

The participants explained that there was clear and continuous communication post the implementation of the system. A participant said:

‘There was a lot of communication regarding the project.’ (P1, female, unit manager)

The participants stated that communication also took place among the employees. There was continuous sharing of relevant information among colleagues:

‘They also heard from other colleagues as the hospital was a pilot.’ (P1, female, unit manager)

The participants indicated that the hospital group communicated through various methods. The participants shared that they were satisfied with the communication methods used. Comments were made that the methods of communication used included posters, leaflets, emails, WhatsApp, and the media:

‘Posters everywhere throughout the hospital.’ (P3, male, registered nurse)

‘Updates on the project were communicated through posters, leaflets and emails for managers to update employees.’ (P5, male, unit manager)

‘Other than receiving communication from the Unit Manager, they were given printed leaflets about the project.’ (P6, female, enrolled nurse)

‘The Unit Manager created a WhatsApp group for ongoing communication about the project.’ (P7, male, enrolled nursing assistant)

‘Ongoing communication through posters and emails.’ (P9, female, anaesthetic registered nurse)

The participants suggested that information was accessible to everyone, as it was displayed in plain sight:

‘The Unit Manager did the communication, and there were posters all over the hospital on the walls.’ (P4, female, registered nurse)

#### Sub-theme 1.1: Breakdown communication

Participants have indicated that there was communication during the implementation of the digitalisation. The participants attest that:

‘Overall, while I believe digitalisation can bring many benefits, the way it’s being implemented here, with poor communication and lack of proper training, is making it more of a hindrance than a help.’ (P12, male, registered nurse)

‘No clear protocol was communicated to us on handling such situations, and management was unreachable then. We ended up reverting to paper records temporarily, which caused confusion and frustration among the staff.’ (P8, female, registered nurse)

### Theme 2: Rationale for electronic medical records system implementation

This theme emerged from participants’ responses regarding whether the reasons behind the decision to implement the new EMR system were effectively communicated. All 10 participants (100%) indicated that both the *project team and management clearly explained* the purpose of the system’s introduction across the hospital group. According to the participants, the primary *rationale for implementation* was to enhance *workflow efficiency* and *streamline patient care processes*. They highlighted that the system has significantly improved *admission, patient hospital stays and discharge procedures*, making these processes faster and more efficient. Elaborating on workflow improvement, The participant stated that:

‘Discharges don’t take long, and the nurses know way in advance which patients will be discharged.’ (P1, female, unit manager)

Medication prescription and ordering, including blood tests, are easier, as all these processes are now carried out on the system. The participants explained that the system is efficient and convenient. The participants further indicated that the system is user-friendly and patient records are easily accessible:

‘Easier access to the patient’s information.’ (P1, female, unit manager)

It is much easier to work on the new EMR system, as the patient’s files and records are on one device, than working on multiple manual files:

‘Working with lots of patients, it is easier to work on one device, and patient’s files are easy to access than manual files.’ (P3, male, registered nurse)

The participants commented that retrieving patients’ past medical data is easy. The participants shared the following:

‘Medical history accessible in the event that there is another admission or follow-up.’ (P1, female, unit manager)

‘Reasons for the system’s implementation included proper record keeping, easy access to patient records and history.’ (P6, female, enrolled nurse)

The nurses can prepare well in advance on the patient’s treatment plans instead of going to each patient’s bedside to flip through files to check the instructions and nursing care procedures:

‘Medication prescriptions and instructions are easier – no mistakes or errors.’ (P2, female, unit manager)

The participants stated that the implementation of EMRs will improve security levels and protect patients’ confidential information. The participants had the following to say:

‘Information stored in a secure platform rather than manual files accessible to unauthorised individuals.’ (P1, female, unit manager)

‘Confidentiality for patient records as there are no files lying around with confidential information accessible by unauthorised individuals.’ (P7, male, enrolled nursing assistant)

‘Password-protected access ensures data privacy.’ (P9, female, anaesthetic registered nurse)

The participants stated that lack of storage was also an issue regarding hard copies of patients’ data. The EMR system will assist in this matter because the medical records of patients will be kept in the system.

### Theme 3: Training and support

This theme emerged when participants were asked about the training and support provided before and after the EMR system implementation.

All 10 participants (100%) confirmed that training was adequate and continuous support was available through a dedicated clinical assistant support team, which provided 24/7 assistance. Participants described how management and the project team ensured that information sessions and workshops were conducted before implementation to familiarise employees with the system:

‘A workshop was conducted three months before implementation to introduce employees to the new system.’ (P7, male, enrolled nursing assistant)

‘Information sessions were held for unit managers before implementation.’ (P9, female, anaesthetic registered nurse)

Participants also expressed satisfaction with the quality and relevance of the training:

‘There were adequate resources in the training; each staff member had their own tablets, even in units that do not typically share devices.’ (P4, female, registered nurse)

‘Training was relevant to the nurses’ scope of practice.’ (P6, female, enrolled nurse)

‘Training was considered adequate, and employees had the opportunity to practice on a demo system.’ (P7, male, enrolled nursing assistant)

‘Sufficient training was provided, with employees working on a demo system during training.’ (P9, female, anaesthetic registered nurse)

While participants acknowledged the efforts made by management and the project team, they also noted that the information provided during training was excessive, making it challenging to retain all details simultaneously.

### Theme 4: Overall perceptions of employees

This theme emerged when participants were asked about their overall experiences with the transition from manual patient records to the new EMR system and their views on change management and system implementation.

The responses revealed mixed emotions among employees. While some excitedly welcomed the new system, others were apprehensive about the change. Ten participants (100%) expressed positive attitudes, emphasising that despite its challenges, they preferred the EMR system over manual records:

‘The nurses were very excited about the project.’ (P2, female, unit manager)

‘They could not wait for the system to be implemented in their ward.’ (P3, male, registered nurse)

‘The system is way better than manual files.’ (P3, male, registered nurse)

However, participants also observed that not all employees shared the same enthusiasm. Some, particularly older nurses, were resistant to the transition, and a few even left their positions because of discomfort with the new system:

‘Some older nurses and doctors left due to the system’s implementation.’ (P7, male, enrolled nursing assistant)

‘Mixed reactions from older-generation nurses, with some feeling uneasy.’ (P8, female, registered nurse)

Anxiety levels were also high, as some employees feared losing data or making errors in a live system:

‘Nurses were very anxious, and the concerns were around loss of information.’ (P1, female, unit manager)

‘Employee anxieties and concerns about making errors on the live system.’ (P10, male, registered nurse)

Overall, the study revealed that while most participants embraced the new system, age and prior experience influenced employees’ comfort levels with the change.

### Theme 5: Technology literacy

This theme emerged when participants were asked about their previous experience with technology and their comfort levels using digital systems. Only five participants (50%) could articulate their computer proficiency levels, highlighting that employees had varying technological experience. Some lacked basic computer skills, particularly older staff members:

‘What should have been considered before implementation was that not all employees had worked on a computer before – older employees did not receive computer training during their nursing education.’ (P9, female, anaesthetic registered nurse)

‘Some employees already had computer experience, facilitating their adaptation to the new system.’ (P7, male, enrolled nursing assistant)

‘Some nurses reported difficulties adapting to the new system on iPads, as they were more familiar with using smartphones for digital interactions. The transition to a different device required additional practice and support.’ (P5, male, unit manager)

Older nurses were particularly concerned about their ability to operate the EMR system, with some fearing job insecurity because of the transition:

‘Concerns among older staff about job security due to technology adoption.’ (P8, female, registered nurse)

Additionally, management-level employees also struggled with computer literacy:

‘I am one of the unit managers with little experience working on computers; my proficiency is at a beginner level as I did not have much computer background.’ (P2, female, unit manager)

Participants further emphasised that low technology literacy negatively affected patient care:

‘For nurses not used to technology, it is taking them a little more time to complete tasks.’ (P2, female, unit manager)

Technology literacy levels varied among the management and the employees. From the responses of the participants, a lack of computer proficiency negatively impacted patient care because they delayed when servicing the patients.

### Theme 6: Limitations of the whole-electronic medical records approach

From the participants’ responses, it was gathered that seven participants (70%) raised concerns about the limitations of the new system.

The participants were concerned about the network challenges that were encountered. The participants explained that long periods of network breakdown were concerning. The manual approach was more convenient in some instances. Participants stated that:

‘There are network issues – sometimes slow.’ (P2, female, unit manager)

‘Quick scanning of records when the system is back online, but practicality concerns during extended offline periods.’ (P7, male, enrolled nursing assistant)

‘Challenges with the system’s speed due to network issues.’ (P10, male, registered nurse)

Elaborating on the convenience of the manual approach:

‘Network problems led to downtime, during which manual records were used and later scanned onto the system.’ (P7, male, enrolled nursing assistant)

When working on the EMR system, the participants explained that communication challenges were experienced. The participants were not notified when new changes were implemented in the system. This is derived from the response below:

‘When the upgrades are done, they don’t get informed timeously; they just see changes when they come on shift.’ (P7, male, enrolled nursing assistant)

The participants explained that the process of digitalisation is expensive. The participants raised the issue of economic efficiency. This is derived from the following response:

‘Understanding that replacing medical equipment can be costly.’ (P6, female, enrolled nurse)

The participants explained that there are risks associated with the EMR system, as there is potential for misinterpretation of automatically recorded vitals:

‘Potential for misinterpretation of automatically recorded vitals.’ (P8, female, registered nurse)

From the participants’ responses, it has been established that the EMR approach has limitations. There are numerous breakdowns of the network, which hinder workflow. The employees are not informed in a timely manner about changes made to the system. Furthermore, replacing medical equipment is costly.

## Discussion

The aim of the study was to explore and better understand nurses’ attitudes and readiness towards implementing an integrated EMR system within a multidisciplinary environment in a private hospital, considering the patient’s journey in an acute hospital setting. The study demonstrated that the new EMR system has significantly eased nurses’ daily management tasks. Key benefits highlighted include the elimination of issues related to the legibility of handwriting, enhanced workflow from admission through hospital stay to discharge, and improved access to patients’ medical records and clinical history. These improvements have streamlined the documentation process, making it more efficient and effective. However, varying levels of computer proficiency among employees indicated that a skills assessment audit could have been beneficial for gauging readiness for the new technology. Despite widespread use of smartphones, navigating a new system on an iPad presented challenges for some nurses. Resources were adequate, with patient records consolidated on a single device and securely stored. Nonetheless, network issues and outdated medical equipment posed significant challenges. Recent studies underscore the complexities of digital transformation in healthcare. Chengoden et al. (2023) explored healthcare workers’ readiness for digital tools, revealing significant concerns about data security, privacy, a lack of training and resistance to change. Tack et al. (2022) surveyed over 1000 HCPs, highlighting barriers despite acknowledging the benefits of digital tools. Addressing these concerns requires targeted training programmes and robust infrastructure. Kinicki and Fugate (2021) emphasise that a supportive organisational culture and leadership are crucial for embracing digital tools. Lampo (2022) concurs, observing the need for clear guidance and champions for innovation among healthcare workers. Hyde et al. (2023) found that involving healthcare workers in shaping digital implementation strategies increased their willingness to accept change. These studies highlight the importance of addressing both technical and cultural issues to improve readiness and willingness to embrace digital tools.

While the existing literature extensively covers the significance of communication, user involvement and training in healthcare technology adoption, these findings provide unique insights into the distinction between challenges and facilitators of digital transformation in a specific healthcare context. One key distinction is the emphasis on the interplay between communication strategies and user readiness. While Fernández-Portillo et al. (2022) and Graf et al. (2023) highlight the importance of clear communication, these findings extend this understanding by demonstrating how tailored communication approaches directly influence enthusiasm for technology adoption. The ability to align messaging with healthcare workers’ expectations and concerns is crucial in determining system acceptance.

In addition, the findings contribute new perspectives on the role of continuous, adaptive training. While prior studies (Iivari, Sharma & Ventä-Olkkonen 2020; Nguyen & Broekhuizen 2022) stress the need for ongoing learning, these findings suggest that initial onboarding gaps, especially in practical, day-to-day applications, are often overlooked. The importance of customised training that accounts for varying levels of technological proficiency (Blut & Wang 2020) is further emphasised, particularly in healthcare environments with diverse staff competencies. Another unique aspect is the exploration of the emotional dimensions of technology adoption. While Bronsoler et al. (2020) discuss resistance because of job displacement fears, these findings highlight a more complex duality: healthcare workers appreciate the benefits of digitalisation but simultaneously experience uncertainty about evolving job roles. This suggests that managing perceptions of role security should be integral to digital transformation strategies.

Furthermore, the findings contribute to the literature by contextualising digital transformation challenges within the private healthcare sector. Many studies focus on public healthcare constraints (Adade Williams, Sikutshwa & Shackleton 2020; Qader et al. 2022), yet these findings demonstrate that even well-resourced private institutions face barriers such as network disruptions, system compatibility issues and workflow integration challenges. The emphasis on technological infrastructure limitations and the need for proactive communication regarding system upgrades (as highlighted by Cetindamar Kozanoglu & Abedin 2021) presents a critical insight for healthcare technology management. These findings offer a refined understanding of digital adoption dynamics, particularly by integrating perspectives on communication, continuous learning, emotional readiness and private-sector-specific challenges. They reinforce the existing knowledge while providing practical considerations for improving digital transformation strategies in healthcare settings.

## Practical implications and conclusion

The study highlights the critical importance of technology adoption and change management in healthcare, particularly within private hospital groups. As the sector transitions from manual health record management to EHRs, improving efficiency, patient care and long-term sustainability becomes imperative. However, this shift requires careful consideration of human factors, such as behaviour, attitudes and readiness. The practical implications of this study highlight the need for a strategic approach to technology adoption, emphasising the benefits of EMR implementation, tailored training and continuous communication. Success in these initiatives hinges on employee engagement, support and management involvement. Additionally, addressing factors such as emotional support, bridging technology literacy gaps, ensuring network stability, providing timely system change notifications, managing costs, mitigating risks and incorporating feedback mechanisms can significantly enhance the implementation process. By following these recommendations, private hospital groups can ensure a smoother transition to EHRs, leading to better patient care, increased efficiency and a stronger position in the competitive healthcare landscape. This comprehensive approach integrates both technological and human elements vital for successful EMR implementation and operation.
